# Clinical efficacy of different approaches for laparoscopic intersphincteric resection of low rectal cancer: a comparison study

**DOI:** 10.1186/s12957-022-02521-5

**Published:** 2022-02-22

**Authors:** Wenquan Ou, Xiaohua Wu, Jinfu Zhuang, Yuanfeng Yang, Yiyi Zhang, Xing Liu, Guoxian Guan

**Affiliations:** 1 Department of General Surgery, Affiliated Nanping First Hospital, Fujian Medical University, 317 Zhongshan Road, Nanping, 353000 Fujian China; 2 Department of Colorectal Surgery, Affiliated First Hospital, Fujian Medical University, 20 Chazhong Road, Fuzhou, 350005 Fujian China; 3 Department of Colorectal Surgery, Fujian Medical University Union Hospital, Fujian Medical University, 29 Xinquan Road, Fuzhou, 350001 Fujian China

**Keywords:** Low rectal cancer, Operative approach, Laparoscopy, Intersphincteric resection, Anal function

## Abstract

**Background:**

The operative results of different approaches for the laparoscopic intersphincteric resection (LAISR) of low rectal cancer vary, and the patient characteristics associated with the best outcomes for each procedure have not been reported. We compared the efficacy of different approaches for LAISR of low rectal cancer and discussed the surgical indications for each approach.

**Methods:**

We retrospectively reviewed data from 235 patients with low rectal cancer treated via LAISR from October 2010 to September 2016. Patients underwent either the transabdominal approach for ISR (TAISR, *n* = 142), the transabdominal perineal approach for ISR (TPAISR, *n* = 57), or the transanal pull-through approach for ISR (PAISR, *n* = 36).

**Results:**

The PAISR and TAISR groups exhibited shorter operation times and less intraoperative blood loss than the TPAISR group. The anastomotic distance was shorter in the PAISR and TPAISR groups than in the TAISR group. No differences in the ability to perform radical resection, overall complications, postoperative recovery, Wexner score recorded 12 months after ostomy closure, 3-year disease-free survival, local recurrence-free survival, distant metastasis-free survival, or overall survival (OS) were observed among the three groups.

**Conclusions:**

TAISR, TPAISR, and PAISR have unique advantages and do not differ in terms of operation safety, patient outcomes, or anal function. TPAISR requires a longer time to complete and is associated with more bleeding and a slower recovery of anal function. PAISR should be considered when TAISR cannot ensure a negative distal margin and the tumor and BMI are relatively small; otherwise, TPAISR is required.

## Background

Abdominoperineal excision of the rectum (APE) was once the dominant procedure for treating low rectal cancer (5 cm from the inferior margin of the anus); however, APE resulted in extensive trauma and permanent colostomy, thereby markedly affecting the receiving patient’s quality of life [1]. In recent years, the exploration of sphincter-preserving surgery for low rectal cancer is full of promise. Even if the tumor invaded the ipsilateral puborectalis muscle, there has been reported to successful sphincter preservation after hemilevator excision [2]. However, many studies seem to be more interested in transsphincterectomy. Intersphincteric resection (ISR) is proposed as an extreme anal sphincter procedure based on total mesorectal excision (TME). By removing some or all of the internal sphincter, a sufficient distal margin is obtained [3, 4]. Multiple studies have confirmed satisfactory oncological outcomes and quality of life after ISR [5, 6].

Many reports have discussed laparoscopic ISR [5, 7–10]. Most of these reports have involved dissection of the intersphincteric space based on the laparoscopic TME technique, which is followed by transection of the rectum through the perineum at a safe distance from the distal edge of the tumor under direct vision, and handsewn repair [9, 11, 12]. This operative approach is complicated and technically difficult, particularly because separation of the anterior rectal wall can easily damage the vagina in women (the urethra and prostate may be damaged in men), and many postoperative complications and severe anal functional damage have been noted [3, 4]. Several investigators have postulated that laparoscopic transabdominal partial ISR is feasible and would also achieve the goal of obtaining a negative distal resection margin [13]. However, because determining the inferior margin of the tumor is limited by the pelvic cavity and use of laparoscopic instruments, laparoscopic transabdominal partial ISR is much more likely to lead to a positive distal margin [14, 15]. Other authors have used the laparoscopic ISR technique to separate the intersphincteric space to the distal position of the pelvic cavity, followed by transanal evisceration of the rectum, to clearly reveal the inferior edge of the tumor. Then, the rectum was transected under direct vision, and instrumented anastomosis was completed using the double anastomosis technique [16]. However, pull-through is difficult in patients with mesenteric fat hypertrophy and relatively large tumors [16]. The premise of all three of these laparoscopic ISR approaches is to separate the intersphincteric space based on laparoscope-assisted TME in order to ensure negative circumferential and distal margins, which is also the crucial and most difficult step of laparoscopic ISR.

However, the relative advantages and disadvantages of these three surgical procedures and the most suitable populations for each have not yet been reported. This article reviews the results of three laparoscopic ISR approaches for low rectal cancer and provides an in-depth discussion with the aim of offering individualized sphincter-preserving solutions for patients with low rectal cancer, while ensuring surgical safety and reducing surgical difficulty and complications.

## Patients and methods

### Patients

Clinical data from 235 consecutive patients with low rectal cancer treated using laparoscopic-assisted ISR radical surgery from October 2010 to September 2016 were retrospectively collected.

Inclusion criteria were as follows: (1) histologically proven rectal adenocarcinoma; (2) tumors located 3–5 cm above the anal edge; (3) mobile tumors detected in a digital rectal examination; (4) tumors that did not involve the external anal sphincter and peripheral structures, as shown by endorectal ultrasound (ERUS), and magnetic resonance imaging (MRI); (5) no evidence of distant metastasis in the preoperative evaluation; (6) patients younger than 70 years of age; and (7) patients with normal anal sphincter function (Wexner fecal continence score < 4).

The exclusion criteria were as follows: (1) patients with preoperative synchronous cancers; (2) lateral lymph node metastases or invasion of the external sphincter, levator ani, or other adjacent organs; and (3) patients who underwent emergency surgery, palliative resection, or lateral lymph node dissection.

The evaluation included a physical examination, colonoscopy with biopsy, endorectal ultrasonography, MRI, and chest CT. Patients with cT3 or node-positive disease received a long course of preoperative chemoradiotherapy (CRT) (5-fluorouracil-based) with a radiation protocol of 45 Gy/25 fractions followed by a 5.4-Gy boost, for a total of 50.4 Gy. However, their right to refuse preoperative chemoradiotherapy was respected. The operation was performed 8–12 weeks after the radiation treatment was complete. All patients underwent ostomy closure at 3 months after the radical operation. The Wexner incontinence score was used to evaluate anal function from the third month onward after ostomy closure. Procedures were performed by the same surgical team. Our team has experience with more than 2000 cases of laparoscopic colorectal surgery. The surgical team selected TAISR, TPAISR, or PAISR based on the patient’s body conformation, pelvic condition, tumor size, tumor site, distance from the inferior margin of the tumor to the anal margin, and the experience of the surgeon. All patients provided written informed consent.

### Surgical procedures

**Fig. 1:**
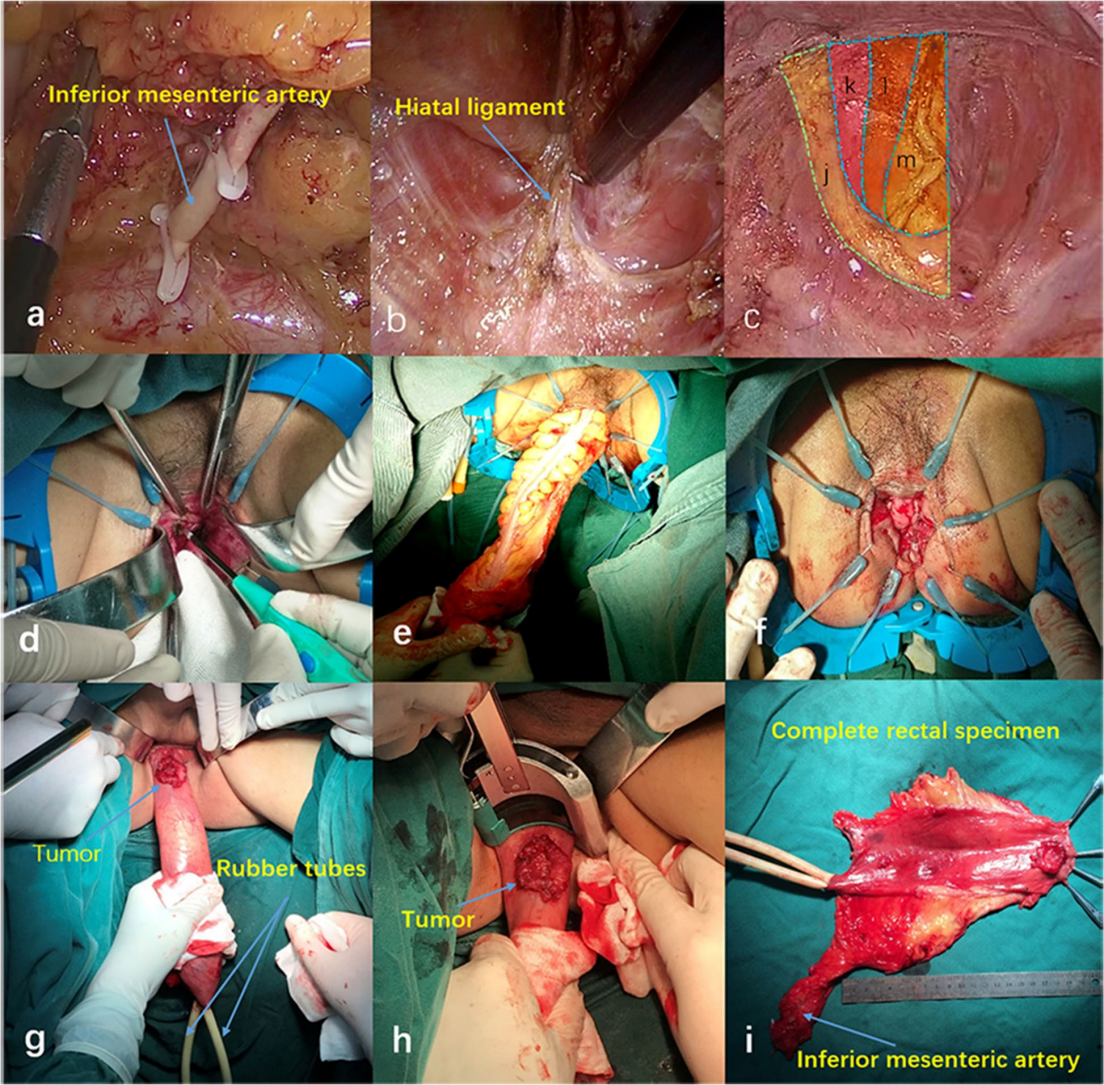
Intraoperative images of the three different LAISR approaches. **a**–**c** Laparoscopic transabdominal approach for intersphincteric resection (TAISR). **a** Inferior mesenteric artery was transected at the root. **b** Hiatal ligament was dissected and transected at the posterior side. **c** Tissue was separated along the puborectalis and internal sphincter to the deep and superficial parts of the external sphincter, and a coloanal anastomosis was performed. J: Puborectalis muscle k: Deep external sphincter muscle l: Superficial external sphincter muscle. m: Rectal stump. **d**–**f** Laparoscopic transabdominal perineal approach for intersphincteric resection (TPAISR). **d** Circumferential incision of the rectal mucosa into the intersphincteric space. **e** Prolapse of the rectum after complete rectal dissociation along the sphincter space. **f** Colonic anal canal or rectal anal canal anastomosis was performed through the perineum. **g**–**i** Laparoscopic transanal pull-through approach for intersphincteric resection (PAISR). **g** Pull-through of the distal rectum with two rubber tubes. **h** Transection of the distal intestine under direct vision. **i** Tumor specimen was intact, and the distal margin was clearly visible

The intraoperative images of the three different LAISR approaches are shown in Fig. 1.

#### Transabdominal approach for ISR (TAISR) (Fig. 1a–c)

Surgery included high ligation of the inferior mesenteric artery and full mobilization of the left colon and the splenic flexure. A standard laparoscopic TME procedure was completed to the levator ani plane. The rectum and its mesentery were separated to the levator ani plane, and the hiatal ligament was exposed with a standard laparoscopic TME procedure. The lower edge of the tumor was determined by digital examination of the anus. The “U” puborectalis muscle was exposed after the hiatal ligament was transected on the posterior side. The line between the puborectalis muscle and the internal anal sphincter was extended, and the tissue was separated to the deep and superficial components of the external sphincter until the level of the dentate line was reached. The anal examination again confirmed that the inferior margin was ≥ 2 cm. The cancer’s lower edge was transected with the stapling device, and the distal margin was observed visually. If unsatisfactory, a specimen of the distal resection margin was sent for frozen sectioning and pathological examination. If the resection margin was clean, anastomosis of the colon and anus was performed with a circular stapler.

#### Transabdominal perineal approach for ISR (TPAISR) (Fig 1d–f)

Abdominal surgery was performed by the transabdominal team. The perineal team exposed the anus, dilated the anal canal up to three horizontal finger widths, and exposed the anal canal with a Lone Star hook to reveal the inferior margin of the tumor. The rectum and anal canal were irrigated with povidone iodine to prevent scatter of cancer cells. Following closure of the anal orifice at least 1 cm below the tumor, the internal sphincter was circumferentially incised and the intersphincteric plane was dissected. The dissection was then carried cephalad to connect with the abdominal team. After the distal rectum was pulled out of the anus, the sigmoid colon was transected 15 cm above the superior tumor margin and specimen was removed. Next, a 5% iodophor solution was used to wash the surgical wound. Ultimately, coloanal anastomosis was performed via a handsewn procedure.

#### Transanal pull-through ***approach*** for ISR (PAISR) (Fig 1h–i)

Abdominal surgery was performed by the same transabdominal team. To excise the rectum, two rubber tubes were inserted through the anus. At 10 cm from the proximal end of the tumor, the two rubber tubes and the rectum were cut together using a linear stapler. Then, the anal side of the two rubber tubes was grasped by hand, and the rectal mucosa was gently pulled out in the anal direction via a laparoscopic operation. This procedure allowed the surgeon to easily pull the rectal stump through. Next, the intestinal cavity was rinsed several times with 0.5% iodophor and distilled water. The tumor was clearly detected, and then the intestine was transected 1–2 cm from distal margin of tumor with a CONTOUR cutter under direct vision. Finally, the rectal stump was returned to the pelvic cavity through the anus, and anastomosis of the intestine was performed using a tubular stapler.

### Statistical analysis

Quantitative data are presented as mean and standard deviation or median and percentile (25th–75th). Qualitative data are presented as absolute numbers and percentages. Categorical data were compared using the Chi^2^ test or Fisher’s exact test. Continuous variable was compared using the Kruskal-Wallis test. Kaplan-Meier estimates were performed for survival analysis, and the difference in survival between groups was tested using the log-rank method. All statistical analyses were performed using SPSS 20.0 software. Differences were considered significant at *P* values < 0.05.

## Results

### Patient and tumor characteristics

**Fig. 2:**
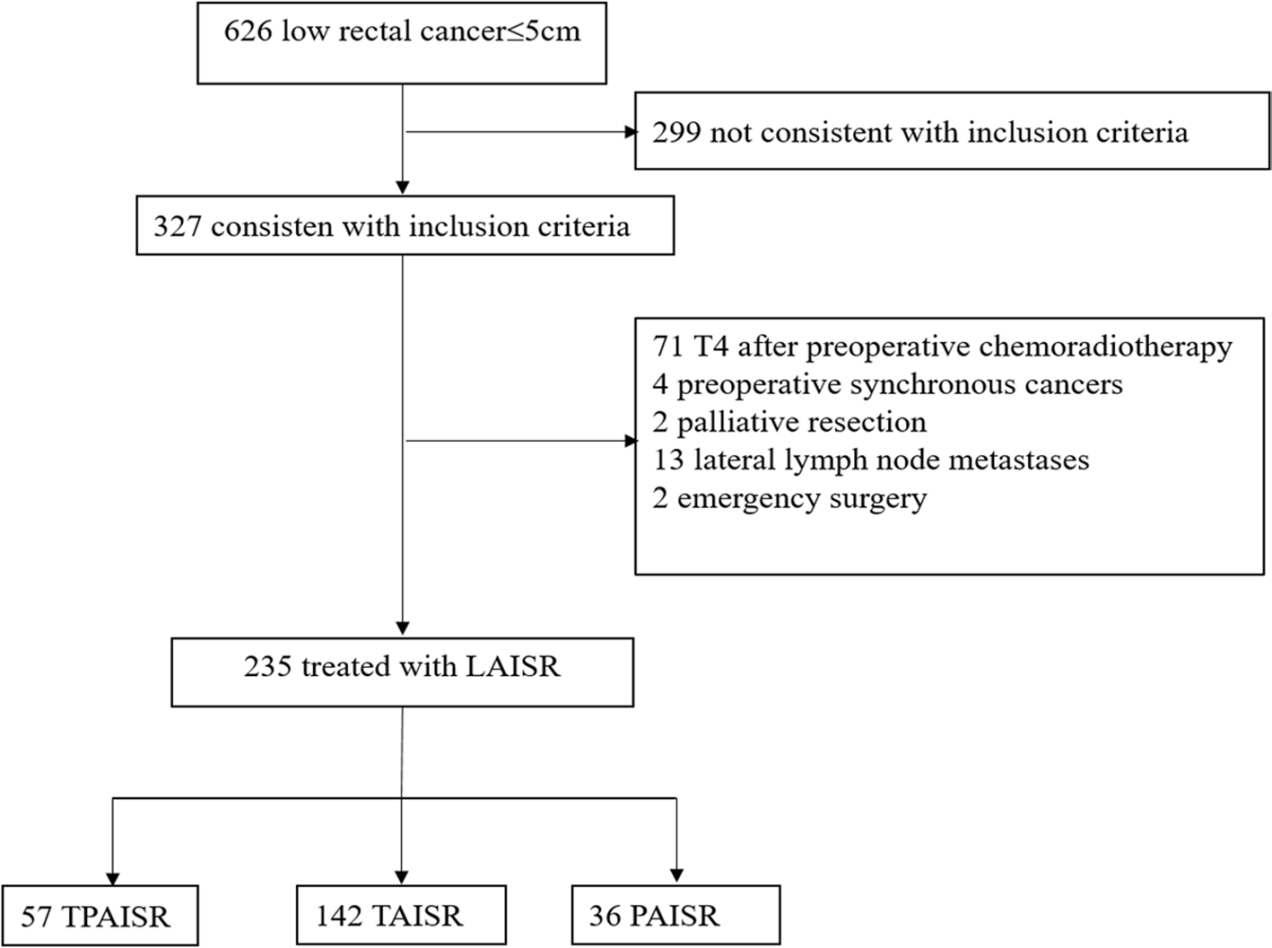
Flow chart of patient selection. *LAISR* laparoscopic intersphincteric resection, *TPAISR* transabdominal perineal approach for ISR, *PAISR* transanal pull-through approach for ISR, *TAISR* transabdominal approach for ISR

A comparison of the characteristics of the 235 patients is provided in Table 1. In total, 142 patients underwent TAISR, 57 underwent TPAISR, and 36 underwent PAISR. Figure 2 shows the patient selection flow chart. Significant differences in gender, age, ASA classification, previous abdominal surgery, tumor size, pre-neoadjuvant therapy, tumor markers (CEA and CA199), neoadjuvant chemoradiation, preoperative T stage, or preoperative baseline Wexner scores were not observed among the three groups (*P >* 0.05). BMI was lower in the PAISR group than in the TPAISR group (21.8 kg/m^2^ vs. 23.2 kg/m^2^, *P* = 0.032). The distance from the inferior margin to the anal margin was smaller in both the PAISR group and the TPAISR group (3.8 cm and 4.0 cm, respectively) than in the TAISR group (5.0 cm) (*P* < 0.001). The proportion of patients with a luminal circumference of the tumor (LCIT) > 50% was significantly smaller in the PAISR group (27.8%) than in the TPAISR group (63.2%) (*P* = 0.001).

**Table 1:** Preoperative characteristics of the patients and tumors

	TPAISR *n* = 57 (%)	TAISR *n* = 142 (%)	PAISR *n* = 36 (%)	*P* value
Gender				0.854
Male	32 (56.1)	76 (53.5)	21 (58.3)	
Female	25 (43.9)	66 (46.5)	15 (41.7)	
Age, median (IQR), years	52	54 (46, 62)	58	0.059
BMI, median (IQR)	23.2	22.2	21.8	**0.032** **0.021**^**a**^ 0.527^b^ **0.024**^**c**^
ASA score				0.223
I	40 (70.2)	89 (62.7)	17 (47.2)	
II	12 (21.2)	43 (30.3)	15 (41.7)	
III	5 (8.8)	10 (7.0)	4 (11.1)	
Tumor height, median (IQR), cm	4.0	5.0	3.8	**< 0.001** **< 0.001**^**a**^ **< 0.001**^**b**^ 0.431^c^
Previous abdominal surgery				0.291
No	48 (84.2)	125 (88.0)	28 (84.2)	
Yes	9 (15.8)	17 (12.0)	8 (22.2)	
Tumor size, median (IQR), cm	2.5	2.5 (1.8, 3.0)	2.0 (2.0, 3.0)	0.440
LCIT (%)				**0.002**
< 50	21 (36.8)	83 (58.5)	26 (72.2)	0.130^b^
≥ 50	36 (63.2)	59 (41.5)	10 (27.8)	**0.001**^**c**^
CEA (ng/ml)				0.272
≤ 5.0	46 (80.7)	110 (77.5)	24 (66.7)	
> 5.0	11 (19.3)	32 (22.5)	12 (33.3)	
CA199 (u/ml)				0.076
≤ 37.0	51 (89.5)	126 (88.7)	27 (75.0)	
> 37.0	6 (10.5)	16 (11.3)	9 (25.0)	
Neoadjuvant CRT				0.111
No	11 (19.3)	48 (33.8)	9 (25.0)	
Yes	46 (80.7)	94 (66.2)	27 (75.0)	
Preoperative T category				0.116
T1	14 (24.6)	40 (28.2)	3 (8.3)	
T2	23 (40.4)	64 (45.1)	21 (58.4)	
T3	13 (35.0)	38 (26.8)	12 (33.3)	
Preoperative baseline Wexner scores	1 (0–3)	1 (0–3)	1 (0–3)	0.681

Data are reported as the median and percentile (25th–75th) or *n* (%)

*CRT* chemoradiotherapy, *BMI* body mass index, *LCIT* luminal circumference of the tumor, *IQR* interquartile range

^a^TPAISR group compared with the TAISR group

^b^TAISR group compared with the PAISR group

^c^TPAISR group compared with the PAISR group

### Comparison of intraoperative and postoperative complications

**Fig. 3:**
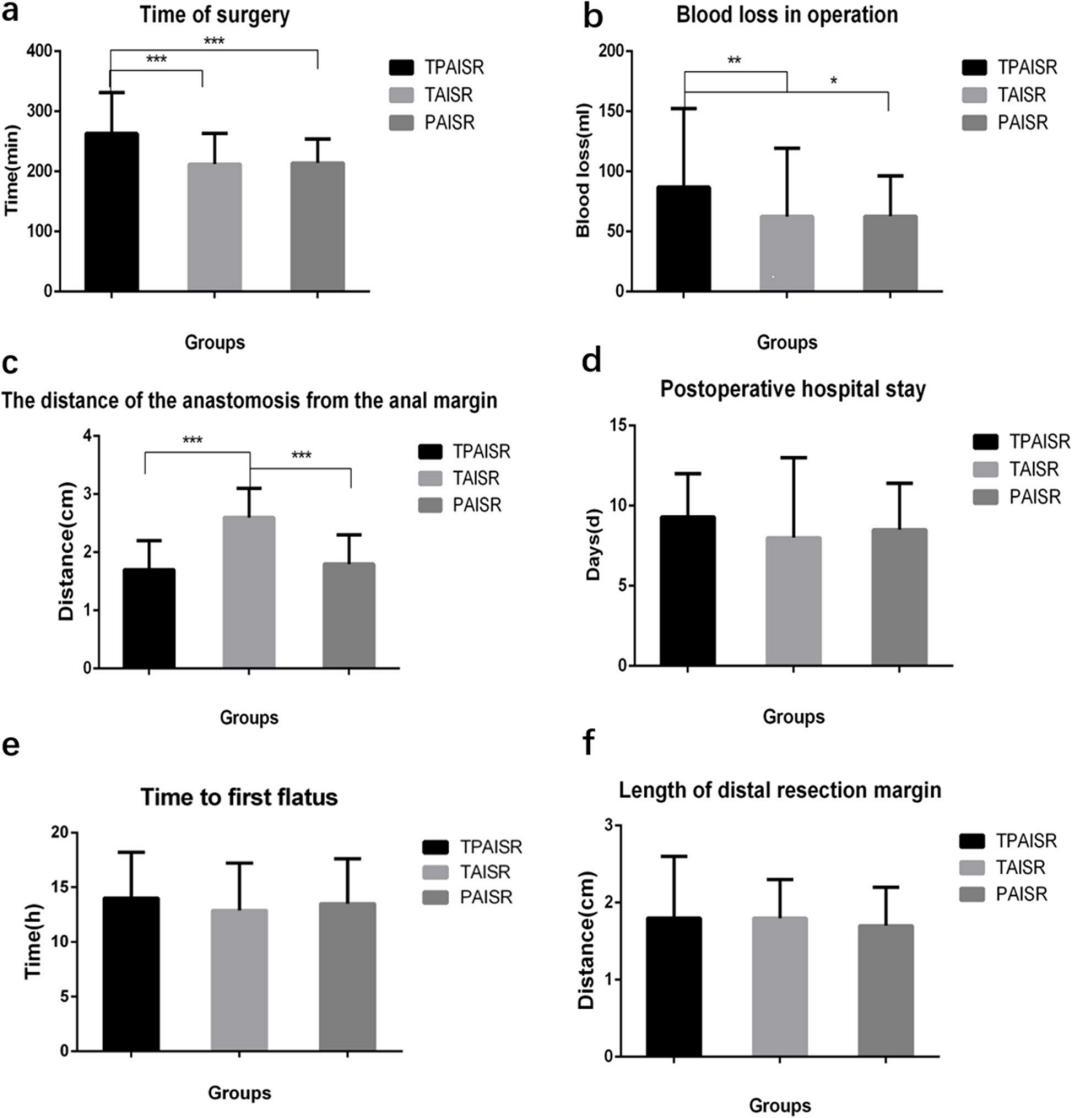
Intraoperative and postoperative indicators. The operation time, intraoperative blood loss, anastomotic distance from the anal margin, length of the distal resection margin, postoperative exhaust time, and postoperative hospital stay were compared. **a** ***P < 0.001 (TPAISR group compared with the TAISR group); (TPAISR group compared with the PAISR group). **b** **P = 0.006 (TPAISR group compared with the TAISR group), *P = 0.045 (TPAISR group compared with the PAISR group). **c** ***P < 0.001 (TPAISR group compared with the PAISR group) and (TAISR group compared with the PAISR group)

Preventive ileostomy was performed in all patients without conversion to laparotomy or death. The operation duration was shorter in the PAISR group (214 ± 40 min) and TAISR group (212 ± 51 min) than in the TPAISR group (263 ± 68 min) (*P* < 0.001) (Fig. 3a). The PAISR group (62.8 ± 33.5 ml) and TAISR group (62.6 ± 56.7 ml) experienced less bleeding than the TPAISR group (86.9 ± 65.3 ml) (*P* < 0.05) (Fig. 3b). The PAISR group (1.8 ± 0.5 cm) was equivalent to the TPAISR group in terms of the anastomotic distance (from the anastomosis to the anal verge) (1.7 ± 0.5 cm) (*P* = 0.360), although the anastomotic distance was greater in the TAISR group (2.6 ± 0.5 cm) (*P* < 0.001) (Fig. 3c).

### Comparison of surgical safety and oncological outcomes

The overall complication rate after ISR was 25.1% (59 cases), including 6 cases (2.6%) of anastomotic leakage (all grade A or B leakage [17]), 6 cases of intestinal obstruction (2.6%), which were cured after non-surgical treatment. No differences in anastomotic leakage, intestinal obstruction, and other surgical related complications were observed among the three groups. The proportions of grade I complications in TPAISR, TAISR, and PAISR groups were 15.8%, 9.2%, and 8.3%, respectively. The rates of grade II complications were 14.0%, 8.5%, 2.8%. Only 1 case of grade III-a complications was observed in the TAISR group. There was no statistical difference in the incidence of grade I-III-a in the three groups. No grade III-b-IV complications occurred in the three groups. No significant differences in safety and radical outcomes were observed among the three groups (*P* < 0.05) (Table 2).

**Table 2:** Comparison of surgical safety and radicalization

	TPAISR *n* = 57 (%)	TAISR *n* = 142 (%)	PAISR *n* = 36 (%)	*P* value
Postoperative complications	17 (29.8)	38 (26.8)	4 (11.1)	0.100
Anastomotic leakage	1 (1.8)	4 (2.8)	1 (2.8)	1.000^e^
Intestinal obstruction	3 (5.3)	3 (2.1)	0 (0.0)	0.341^e^
Anastomotic stenosis	1 (1.8)	1 (0.7)	0 (0.0)	0.636^e^
Incision infection	0 (0.0)	1 (0.7)	0 (0.0)	1.000^e^
Others	12 (21.1)	29 (24.4)	3 (8.3)	0.227
Clavien-Dindo Complication grade				
Grade I	9 (15.8%)	13 (9.2%)	3 (8.3%)	0.413
Grade II	8 (14.0%)	12 (8.5%)	1 (2.8%)	0.192
Grade III				
III-a	0	1 (0.7)	0	1.000 ^e^
III-b-IV	0	0	0	NA
Positive distal margin	1 (1.8)	2 (1.4)	1 (2.8)	0.785^e^
CRMI	2 (3.5)	2 (1.4)	0 (0.0)	0.352^e^
Distal cutting margin (cm)	1.5 (1.5, 1.9)	1.5 (1.5, 2.5)	1.5 (1.0–1.5)	0.065
Lymph nodes harvested, median (IQR)	16 (11, 20)	15 (12, 20)	16 (12, 20)	0.829
Histological differentiation				0.042^e^
Well	0 (0.0)	1 (0.7)	3 (8.3)	
Moderate	57 (100)	139 (97.9)	33 (91.7)	
Poor	0 (0.0)	2 (1.4)	0 (0.0)	
pT category				0.140
T0	9 (15.8)	25 (17.6)	8 (22.5)	
T1	9 (15.8)	17 (12.0)	1 (2.8)	
T2	22 (38.6)	53 (37.3)	21 (58.3)	
T3	17 (29.8)	47 (33.1)	6 (16.7)	
pN category				0.502^e^
N0	42 (73.7)	99 (69.7)	37 (75.0)	
N1	12 (21.1)	38 (26.8)	6 (16.7)	
N2	3 (5.3)	5 (3.5)	3 (8.3)	
pTNM stage^a^				0.054^e^
0	8 (14)	20 (14.1)	8 (22.2)	
I	23 (40.4)	54 (38.0)	18 (52.8)	
II	11 (19.3)	25 (17.6)	0 (0.0)	
III	15 (26.3)	43 (30.3)	9 (25.0)	
Hospitalization, days	8.0 (7.0, 9.0)	9.0 (8.0, 9.0)	8.0 (7.0, 8.0)	0.066

Data are reported as the median and percentile (25th–75th) or *n* (%); ^e^ Fisher’s exact test

*CRMI* circumferential resection margin involvement, *IQR* interquartile range

^a^UICC classification

### Evaluation of postoperative anal function

**Fig. 4:**
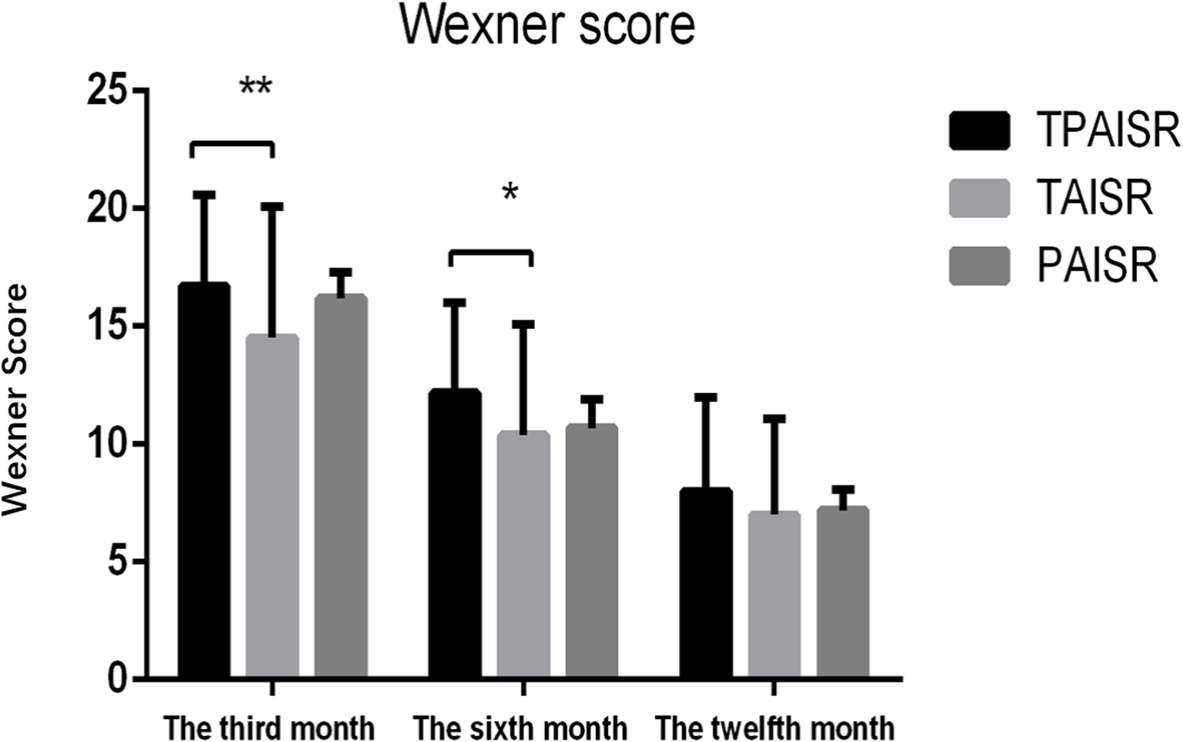
Anal function. Comparison of the Wexner scores of anal function in the 3rd, 6th, and 12th months after ostomy closure among the three groups; *P = 0.008 (TPAISR group compared with the TAISR group); **P = 0.013 (TPAISR group compared with the PAISR group)

The distribution of anal function in the three groups is listed below (Fig. 4). Ninety-one percent of all patients after ISR underwent ostomy closure. In addition, 47 patients, 119 patients, and 30 patients in the TPAISR, TAISR, and PAISR groups were followed up respectively. In the 3rd month after ostomy closure, the Wexner score of the TAISR group (14.5 ± 5.6) was significantly lower than the TPAISR group (16.7 ± 3.9) (*P* = 0.008), but was not different from the PAISR group (16.2 ± 1.1) (*P* > 0.05). In the 6th month after ostomy closure, the Wexner score of the TAISR group (10.4 ± 4.7) was still significantly lower than the TPAISR group (12.2 ± 3.9) (*P* = 0.013), but similar to the PAISR group (10.7 ± 1.2) (*P* > 0.05). In the 12th month after ostomy closure, the Wexner scores of the three groups were 8.0 ± 4.0, 7.0 ± 4.1, and 7.2 ± 0.9, respectively. The anal function scores of the three groups were similar (*P* > 0.05). Seven patients (3.0%) (5 treated with TPAISR) were unable to tolerate poor anal function and underwent a permanent colostomy.

### Comparison of intermediate-term oncological results

**Fig. 5:**
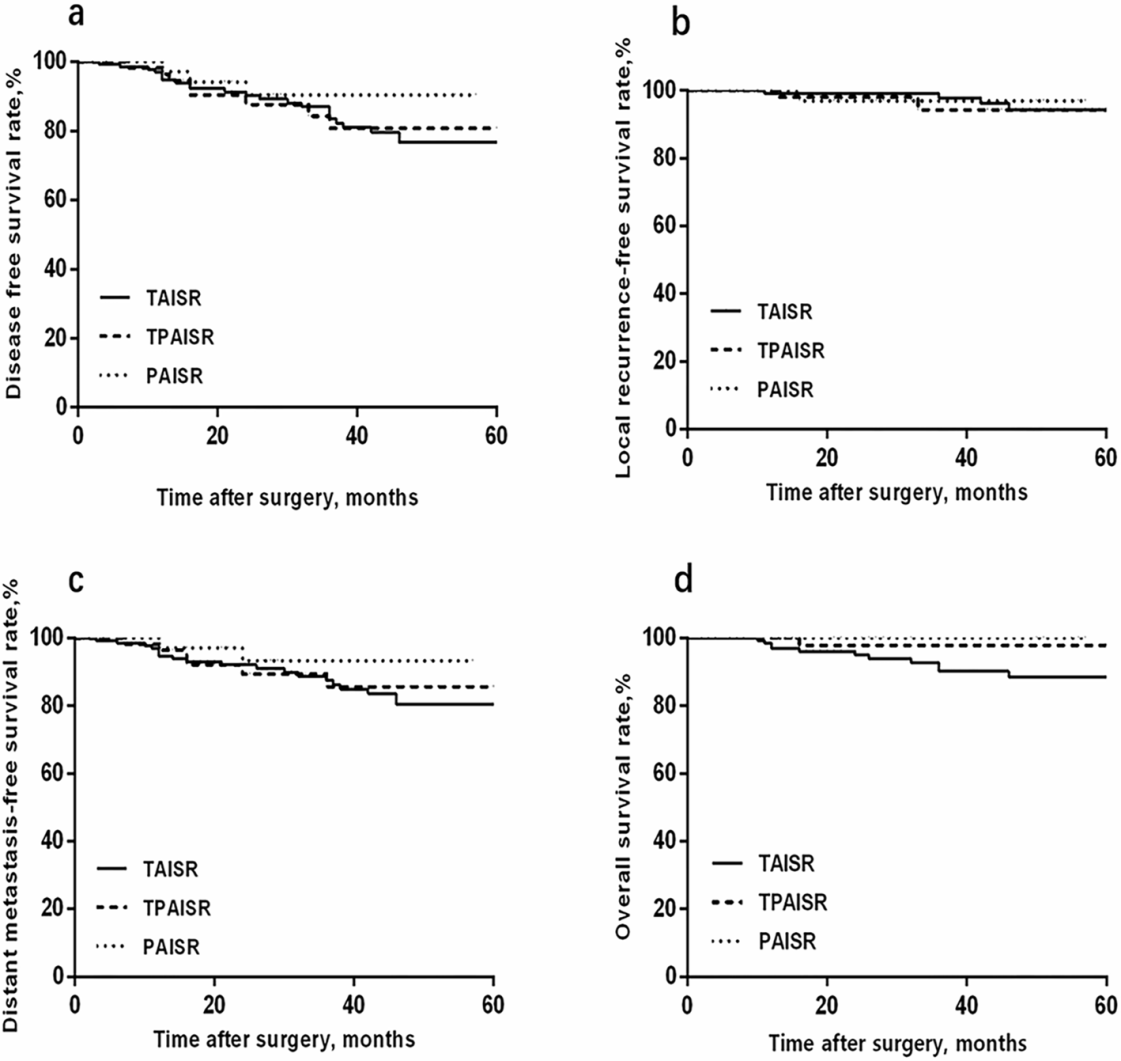
Comparison of intermediate-term oncological results. Comparison of the 3-year DFS (**a**), LRFS (**b**), DMFS (**c**), and OS (**d**) between the three groups. Significant differences in the tumor prognostic indicators listed above were not observed among the three groups (*P* > 0.05)

The median duration of follow-up was 32 months (range 10–77 months), and 96% (226 cases) of the patients underwent follow-up. Ten patients in the TAISR group died within 3 years after surgery: 6 died of distant metastasis, 3 died of unknown causes, and 1 died of local recurrence. One patient in the TPAISR group died due to distant metastasis, and no deaths occurred in the PAISR group. Among the TPAISR, TAISR, and PAISR groups, the 3-year disease-free survival (DFS) (79%, 83.4%, and 81.8%, respectively) (Fig. 5a), local recurrence-free survival (LRFS) (93.7%, 97.7%, and 90.0%, respectively) (Fig. 5b), distant metastasis-free survival (DMFS) (84.3%, 86.6%, and 90.9%, respectively) (Fig. 5c), and overall survival (OS) (97.1%, 89.4%, and 100%, respectively) (Fig. 5d) were not significantly different (*P* > 0.05).

## Discussion

Laparoscopic ISR preserves the anus due to the technique’s ability to dissect the tissue to the rectum, a lower point than that allowed by traditional open ISR, and thus protects both urinary and sexual functions [11]. Despite controversy, many studies have confirmed the feasibility of this technique and have reported satisfactory oncological results as well as acceptable anal function [5, 12, 18]. Based on current evidence, most scholars prefer laparoscopic ISR combined with transperineal tumor resection and manual anastomosis [19, 20], which is called TPAISR. Several scholars have postulated that laparoscopic ISR combined with a double stapler can achieve TAISR without manual anastomosis, is simple to perform, and reduces complications [13]. Several scholars have reported that laparoscopic ISR combined with the pull-through approach removes the difficulty of judging the distal margin, resulting in anal function similar to that observed after low anterior resection (LAR) [16]. Choosing between these three approaches is a challenge for the surgeon.

A method for choosing the most appropriate LAISR surgical approach based on the individual patient characteristics has not been published, and the indications for different approaches for LAISR remain controversial. Fukunaga [21] considered the pull-through technique more suitable for early-stage tumors, although pull-through anal procedures are difficult in patients with lymph node metastasis. In the PAISR group, some of the tumors presented with more than 50% LCIT and T3 stage. This difference in characteristics may be related to the selection bias of this retrospective study and the exploration phase of the PAISR procedure. Furthermore, height from anastomosis level to anal verge was similar in both the PAISR and TPAISR groups, whereas the distance from the lower margin of the tumor to the anal verge in the PAISR group was significantly shorter than that in the TAISR group, but equivalent to that in the TPAISR group. Compared with TAISR, PAISR is considered to perform lower level anastomosis. Therefore, when the TAISR surgeon suspects that the distal margin is insufficient, PAISR may be considered instead. Kwok and Andreola et al. [22, 23] reported that only 4–10% of patients presented with cancer cells spread more than 1 cm to the distal rectum, and only 2% with distal rectal cancer spread 2 cm to the distal end. According to Bujko [24], a distal margin of < 1 cm or > 1 cm does not significantly affect the local recurrence and OS rates. In this study, the average length of the distal margin in the three groups was greater than 1 cm. Compared with the TAISR group, the distance from the anal margin of the tumor was shorter in the PAISR and TPAISR groups, although the distal margin length was similar to that in the TAISR group. PAISR may be safe and suitable for patients with lower rectal cancer, and has advantages in terms of duration of surgery and blood loss.

In the literature, the reported incidence of ISR postoperative complications was 25% on average, of which the incidence of anastomotic leakage was 9.1% and the incidence of anastomotic stenosis was 2.7% [25]. The total complication rate after ISR in this study was 25.1%, including 11.1% in the PAISR group, 29.8% in the TPAISR group, and 26.8% in the TAISR group. No significant differences were noted among the three groups. The anastomotic leakage rates in the three groups (1.8%, 2.8%, and 2.8%, respectively) were lower than some previously reported rates, but similar to the rates reported by Chi et al. [13]. One patient was readmitted for anastomotic leak in the TAISR group. The patient presented only pelvic abscess and could be treated satisfactorily by transperineal US-guided drain without needing a relaparoscopy postoperatively for peritonitis. Although no delayed anastomotic leakage was found in this study, it was reported by Masayoshi et al. [26] so vigilance is still needed during follow-up. In the present study, the anastomotic stenosis rate in all groups was 0.9%, which is lower than previously reported values. The reason may be to mobilize of splenic flexure, remove the irradiated sigmoid colon near the tumor to the greatest extent possible, and descending colon-rectum (anal canal) anastomosis was performed. Only two cases presented with membranous stenosis of the anastomosis, which was improved after regular anal dilation during adjuvant chemotherapy. The rates of surgical site infections (SSI) in this study was low, similar to the results reported by Zheng et al. [27]. In other complications, a significant difference in the incidence of urinary retention was not observed among the three groups, indicating that protection of pelvic floor nerves did not differ significantly among the three ISR approaches.

In the present study, significant differences in the distal resection margin (DRM)-positive rate (1.8%, 1.4%, and 2.8%, respectively, *P* = 0.758) and circumferential resection margin (CRM)-positive rate (3.5%, 1.4%, and 0%, respectively, *P* = 0.352) were not observed among the three groups. The overall DRM-positive rate was lower than that of previous reports (2.8–3.6%) [11, 18, 25], and the overall CRM-positive rate was similar to that of previous reports (2.3–6.2%) [19, 25, 28]. However, one patient in the PAISR group presented with a positive DRM. The male patient had a strong anal sphincter and a small pelvis, with a BMI of 24.4 (kg/m^2^) and LCIT > 50%. The intraoperative abdomen had been separated to an extreme extent, although the pull-through effect was not satisfactory. Therefore, patients with a narrow pelvis, high BMI, and larger tumor may not be suitable for PAISR, and the TPAISR approach should be employed to ensure a negative distal margin. In this study, postoperative pathology of samples from the TAISR group revealed two cases of poorly differentiated adenocarcinoma that possibly resulted from insufficient assessment of the biopsied specimen in the preoperative and postoperative pathological diagnoses.

The local recurrence rate of rectal cancer after ISR ranges from 2 to 13.3% [16, 29]. ISR does not increase the postoperative local recurrence rate of rectal cancer [30]. Previous studies [31–33] have reported a 3-year DFS rate of 67–82.6%, a 3-year OS rate of 81–92.8%, and a 3-year LRFS rate of 82.7–87%. In the present study, the median follow-up time was 32 months. No significant differences in 3-year DFS (79%, 83.4%, and 81.8%, respectively), 3-year LRFS (93.7%, 97.7%, and 90%, respectively), 3-year DMFS (84.3%, 86.6%, and 90.9%, respectively), and 3-year OS (97.1%, 89.4%, and 100%, respectively) were observed among the three groups. This study reported similar intermediate-term results for the three surgical approaches. A higher 3-year OS rate was recorded for the PAISR group, possibly because this group included fewer patients. The low DRM-positive rate and better long-term survival outcomes compared with previous reports reflected the importance of accumulated experience, a specialized team, careful patient selection for the operation, and application of neoadjuvant CRT.

**Fig. 6:**
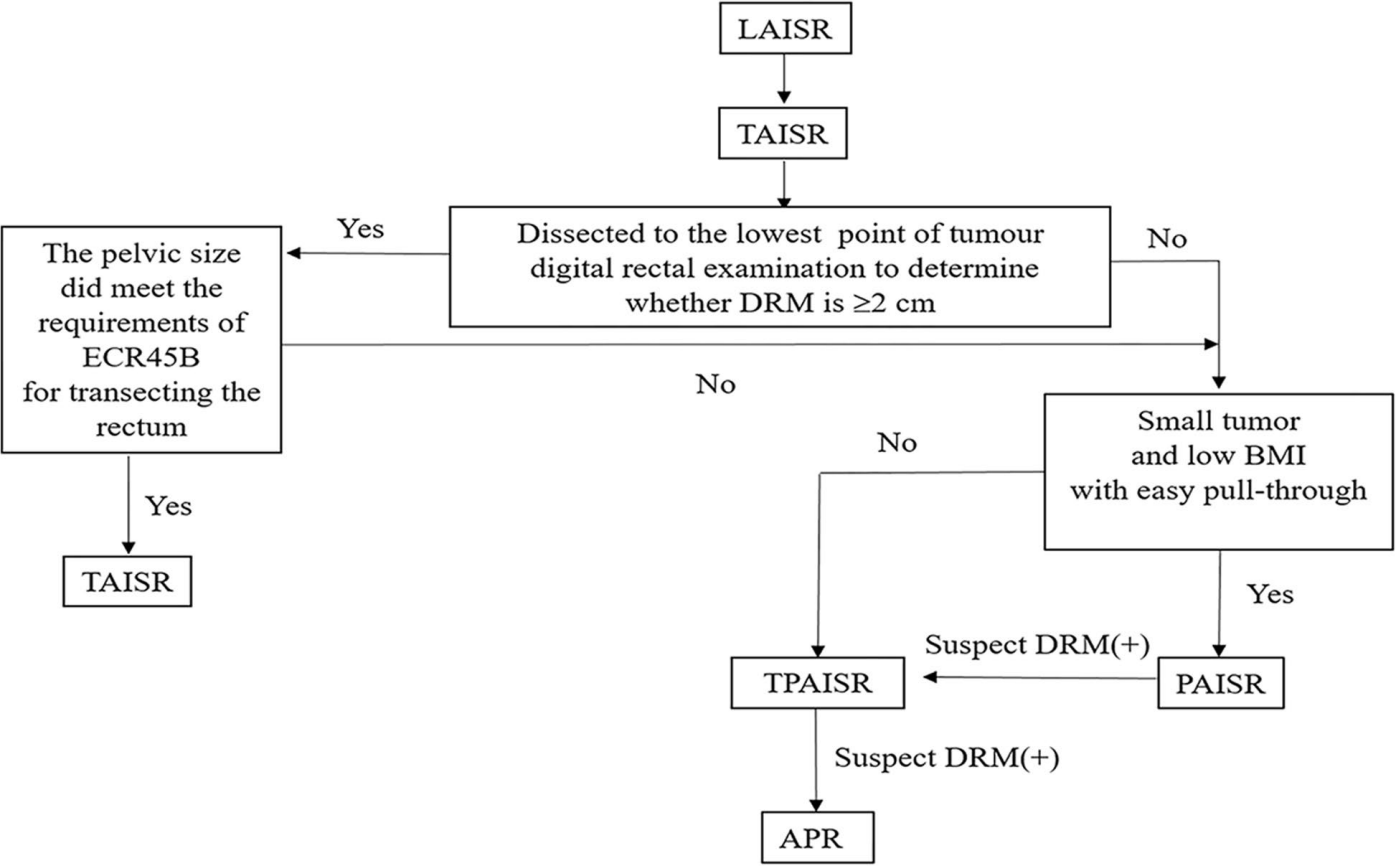
Intraoperative decision flowchart. *LAISR* laparoscopic intersphincteric resection, *TPAISR* transabdominal perineal approach for ISR, *PAISR* transanal pull-through approach for ISR, *TAISR* transabdominal approach for ISR, *APE* abdominoperineal excision of the rectum, *DRM* distal resection margin, *BMI* body mass index

Anal function was poor in the short term after ISR, but satisfactory in the long term. In this study, the Wexner score improved significantly after 12 months compared with the score recorded in the third month, which was consistent with the study by Ito [31]. Anal function was worse in the TPAISR and PAISR groups than in the TAISR group during the first 6 months after ostomy closure. A potential explanation is that the anastomotic distance was lower than the anal margin in the TPAISR and PAISR groups, less rectal mucosa remained. However, a significant difference in anal function was not observed among the three groups at the 12th month after ostomy closure, possibly due to more time to recovery of the anal sphincter caused by the stoma. Second, after levator ani training and biofeedback treatment, “low anterior resection syndrome” was restored to satisfactory results within 1 year [34]. However, some patients are still unable to tolerate anal incontinence, and undergo permanent colostomy [35]. During the follow-up period, seven patients (3.0%) (five of whom underwent TPAISR) underwent permanent colostomy. Thus, the anal function of patients in the TPAISR group recovers slowly in the early stage, and a small number of patients are not satisfied with long-term anal function. Therefore, TPAISR should be considered only when PAISR is unable to be completed or when TAISR is unable to guarantee a negative distant margin. Figure 6 shows the intraoperative decision flowchart. More careful intraoperative management, biofeedback therapy, and careful patient selection may help improve anal function outcomes [13].

This investigation is a retrospective study, and selection bias is inevitable. The low CRMI rate cannot be ruled out caused by selection bias. The low rate of SSI may be related to the use of retrospective data, which requires further measurement with a standard SSI evaluation system. In addition, assessing anal function based solely on the Wexner score is another limitation of this retrospective study. Consequently, further follow-up and randomized controlled trials may lead to more scientific conclusions. In this study, a small number of tumors with a distance of 5 cm from the anal verge were included in the TAISR group; therefore, the results of this group may be similar to those of patients with median rectal cancer. Unfortunately, this study failed to evaluate sexual function, which will be reported in future investigations.

## Conclusions

In conclusion, LAISR (TAISR, TPAISR, and PAISR) of the three different approaches are all optional surgical methods, each with its own advantages, and there is no significant difference in surgical safety, tumor outcome, and anal function. However, TPAISR requires a longer duration of surgery and results in heavy bleeding and slower recovery of anal function. When the TAISR approach is unable to ensure that the distal margin is negative and the tumor and BMI are small, surgeons should consider switching to PAISR; otherwise, they should switch to TPAISR. Based on the individual characteristics of the patient before surgery, a reasonable ISR surgery strategy can be developed, and the surgical approach can be adjusted immediately according to the intraoperative situation, which will maximize the benefit to the patient.

## Data Availability

Some or all data used during the study are available from the corresponding author by request.

## Abbreviations

IRB: Institutional Review Board
ISR: Intersphincteric resection
LAISR: Laparoscopic intersphincteric resection
TAISR: Transabdominal Approach For ISR
TPAISR: Transabdominal Perineal Approach For ISR
PAISR: Transanal Pull-through Approach For ISR
OS: Overall survival
BMI: Body mass index
APE: Abdominoperineal excision
TME: Total mesorectal excision
ERUS: Endorectal ultrasound
MRI: Magnetic resonance imaging
CRT: Chemoradiotherapy
LCIT: Luminal circumference of the tumor
DFS: Disease-free survival
LRFS: Local recurrence-free survival
DMFS: Distant metastasis-free survival
IQR: Interquartile range
SSI: Surgical site infections
